# Multi-Scale Shape Adaptive Network for Raindrop Detection and Removal from a Single Image

**DOI:** 10.3390/s20236733

**Published:** 2020-11-25

**Authors:** Hao Luo, Qingbo Wu, King Ngi Ngan, Hanxiao Luo, Haoran Wei, Hongliang Li, Fanman Meng, Linfeng Xu

**Affiliations:** School of Information and Communication Engineering, University of Electronic Science and Technology of China, Chengdu 611731, China; haoluo@std.uestc.edu.cn (H.L.); knngan@uestc.edu.cn (K.N.N.); lhx@std.uestc.edu.cn (H.L.); hrwei@std.uestc.edu.cn (H.W.); hlli@uestc.edu.cn (H.L.); fmmeng@uestc.edu.cn (F.M.); lfxu@uestc.edu.cn (L.X.)

**Keywords:** shape adaptive network, raindrop and raindrop-free images, raindrop detection and removal, occluded region filtering, clean background preservation

## Abstract

Removing raindrops from a single image is a challenging problem due to the complex changes in shape, scale, and transparency among raindrops. Previous explorations have mainly been limited in two ways. First, publicly available raindrop image datasets have limited capacity in terms of modeling raindrop characteristics (e.g., raindrop collision and fusion) in real-world scenes. Second, recent deraining methods tend to apply shape-invariant filters to cope with diverse rainy images and fail to remove raindrops that are especially varied in shape and scale. In this paper, we address these raindrop removal problems from two perspectives. First, we establish a large-scale dataset named RaindropCityscapes, which includes 11,583 pairs of raindrop and raindrop-free images, covering a wide variety of raindrops and background scenarios. Second, a two-branch Multi-scale Shape Adaptive Network (MSANet) is proposed to detect and remove diverse raindrops, effectively filtering the occluded raindrop regions and keeping the clean background well-preserved. Extensive experiments on synthetic and real-world datasets demonstrate that the proposed method achieves significant improvements over the recent state-of-the-art raindrop removal methods. Moreover, the extension of our method towards the rainy image segmentation and detection tasks validates the practicality of the proposed method in outdoor applications.

## 1. Introduction

Due to the raindrops adhered to a glass window or camera lens, the images captured in rainy weather suffer from poor visibility, which poses significant risks to many outdoor computer vision tasks, such as pedestrian detection [1,2], crowd counting [3], and person re-identification [4]. Therefore, removing raindrops from rainy images is highly desirable, especially in complicated outdoor scenes.

Previous studies on rain removal have achieved great progress and have mainly focused on rain streaks [5,6,7,8,9,10] and rain mist [11,12]. Since the image formation and physical properties of raindrops are very different from those of rain streaks and rain mist, previous methods cannot be applied directly to raindrop removal. Intuitively, raindrops typically show distinct characteristics and complex changes in several aspects, which bring great challenges for removing raindrops while preserving image details. First, due to the diversity of contact surfaces [13], raindrops usually present diverse changes in shape, scale, and direction. Second, due to the different transparency levels, the visibility of regions occluded by raindrops is inhomogeneous, and the image content seen through raindrops may not belong to the areas blocked by the raindrops. Moreover, the movement of raindrops [13] depends not only on the affinity of the surfaces but also the fusion between different raindrops, which is rather than rain streaks falling along specific directions [14].

Recently, raindrop removal has drawn great attention due to its great practicality and challenges. Benefiting from the temporal correlation between consecutive frames, video-based deraining methods [15,16,17] can achieve significant improvements. However, these methods are difficult to extend to common situations where only a single image can be available. To the best of our knowledge, the explorations into single image raindrop removal are still limited in two ways.

On the one hand, the publicly available raindrop image datasets are very limited, in which the diversity and scale of raindrops are insufficient to cover real-world scenes. In [18], Eigen et al. collected 6.5 million 64 × 64 raindrop image patch pairs to train a deep learning-based [19,20] deraining network. Although the raindrop images in [18] were sufficient, the diverse distribution of the raindrop model was relatively poor. For example, most raindrops on the images were particularly small and sparse, thereby failing to effectively occlude the background and provide a distinct refractive effect. In contrast, Qian et al. [21] constructed 861 raindrop image pairs for training, containing larger and more dense raindrops. Regardless of the misalignment between raindrop and raindrop-free regions, the dataset proposed in [21] was not sufficient to cover diverse raindrops and background scenes, even when ignoring the collision and fusion between adjacent raindrops. In addition, the pairs of raindrop and raindrop-free images in [18,21] were obtained by spraying water on glass and then taking photographs of multiple scenes with and without the occluded glass. Thus, this strategy of collecting raindrop datasets is very time-consuming and expensive.

On the other hand, many recent deraining methods have ignored the complex changes in shape, scale, and transparency of raindrops, and applied shape-invariant filters to the whole image, which limited the ability to cope with diverse raindrops. Wu et al. [22] first generated a saliency map to locate small locally salient droplets that only exist in a region of interest (ROI) and then utilized image inpainting techniques to remove the raindrops. Eigen et al. [18] trained a specialized convolution network with constructed training data to remove small raindrops and dirt. Qian et al. [21] designed a generative adversarial network (GAN) combining raindrop images and corresponding attention maps, which helped the model better focus on the raindrop areas. However, they may neglect the complexity of raindrop changes, such as the fusion between large raindrops.

In this paper, to resolve single image raindrop removal problems, we first establish a large-scale synthetic raindrop dataset via automatic rendering, which contains 11,583 high-resolution raindrop and raindrop-free image pairs with diverse backgrounds. Moreover, the raindrops in the proposed dataset present different shapes, scales, and transparency levels, similar to real-world scenes. Second, we propose a Multi-scale Shape Adaptive Network (MSANet) consisting of two branches for detecting and removing diverse raindrops. Specifically, we integrate the receptive field block (RFB) into the detection branch to strengthen raindrop feature extraction and estimate a pixel-level raindrop map for accurately locating raindrops with various shapes and transparency levels. In the removal branch, to capture more texture details for better restoration, we adopt a multi-scale dilated convolution module (MDCM) and multi-scale densely enhanced deformable module (DEDM) to improve the adaptation to complex semantics and diverse raindrops, respectively. Lastly, the final derained result is obtained by fusing the derained output (in the removal branch) and the input raindrop image using the raindrop map (in the detection branch). This can remove diverse raindrops in raindrop regions while preserving the image details in non-raindrop regions. The results demonstrate that the proposed method achieves superior performance over recent state-of-the-art methods.

The detailed contributions of this paper are summarized in the following points:

- First, we extend an automatic raindrop rendering method and construct a large-scale synthetic raindrop dataset including 11,583 high-resolution raindrop and raindrop-free image pairs covering a wide variety of raindrop characteristics and background scenarios.
- Second, we propose a novel end-to-end raindrop removal network called Multi-scale Shape Adaptive Network (MSANet), which is composed of raindrop detection and removal branches. The MSANet can remove raindrops effectively while preserving more image details.
- Third, in the raindrop detection branch, the receptive field block (RFB) is used to strengthen the raindrop feature discriminability for accurately generating a raindrop map. Meanwhile, in the removal branch, the multi-scale dilated convolution module (MDCM) and multi-scale densely enhanced deformable module (DEDM) are adopted to effectively extract semantic information and adaptively remove diverse raindrops, respectively. The final derained result is obtained via a fusion between the two branches for better deraining.
- Lastly, we perform extensive experiments to evaluate the proposed method on both synthetic and real-world raindrop images. The results demonstrate that our proposed method outperforms the recent state-of-the-art methods. Furthermore, the extension of this model to rainy image segmentation and detection can benefit outdoor applications.

## 2. Related Work

### 2.1. Rain Streak and Rain Mist Removal

In general, early methods [23,24,25,26] removed rain streaks in images by formulating rain streak removal as a decomposition model and designing different hand-crafted priors. Under the assumption of a nonlinear composite model between the rain layer and deraining image layer, Luo et al. [25] approximated the patches of two layers using very high discriminative codes with sparsity based priors. Zhu et al. [26] proposed a joint optimization process on a rain-free background layer and rain-streak layer to remove rain-streak details and non-streak details, respectively. When the convolution neural network (CNN) was introduced to single image deraining, learning-based methods were directly used to model and estimate the negative residuals combined with the input rainy image to obtain the derained image. Meanwhile, some researchers [11,12] began to remove rain mist from a single image guided by scene depth information. They often formulated the rain mist as a combination of a single layer rain streak and multi-layer rain accumulation, also known as mist.

### 2.2. Raindrop Removal

#### 2.2.1. Multi-Image Based Raindrop Removal

Roser et al. [15] combined image registration results with accurately detected raindrop positions to restore the occluded regions with intensity information from neighboring image frames. You et al. [16] exploited the local spatio-temporal derivatives of raindrops in videos to separately remove raindrops in partially and completely occluded areas. With long range trajectories, You et al. [17] discovered the motion and appearance features of raindrops locally along the trajectories and then utilized the indicated patches to remove raindrops. Although these video-based methods achieved promising results via temporal information, they are difficult to apply to the common situations where only a single image is available.

#### 2.2.2. Single-Image Based Raindrop Removal

Removing raindrops from a single image often suffers from more challenges than utilizing multiple images. In [18], Eigen et al. built shallow convolution architecture with only three layers to remove raindrops or dirt from degraded images. Although the authors in [18] improved the performance of degraded image restoration, particularly in sparse and small raindrops or dirt, this method tends to lose effectiveness with dense and large raindrops, as shown in [21]. To alleviate this problem, Qian et al. [21] proposed a generative adversarial network (GAN) for raindrop removal. This GAN-based method first adopted a recurrent network combined with convolution LSTMs to produce a raindrop attention map, which indicated the distribution of the raindrops. Then, the generated attention map and the raindrop image were concatenated into the CNN architecture to obtain the final derained result. Despite the network limitations in [18], the authors in [18] and [21] adopted fixed sampling filters and neglected more complex raindrop scenes (e.g., those with collision and fusion between raindrops) in the real world.

### 2.3. Deformable Convolution

The deformable convolution operation [27] was first proposed to extend the original spatial sampling locations in regular convolution and capture information beyond the invariant filtering neighborhood using additional offsets. This makes it widely used in a variety of vision tasks, such as object detection [28], semantic segmentation [27], crowd counting [29], and video super-resolution [30,31,32]. Specifically, Zhang et al. [28] designed a location-aware deformable mechanism to extract the unevenly distributed context features for better offsets estimation. Guo et al. [29] utilized the deformable CNN operation to generate a high-quality density map and accurately predict the counting numbers. Both Wang et al. [30] and Tian et al. [31] adopted deformable convolution as a basic operation for temporal feature alignment. As far as we know, our proposed densely enhanced deformable module is the first method that attempts to incorporate deformable convolution sampling into single image deraining for adaptively removing raindrops with diverse granularity.

## 3. RaindropCityscapes Dataset

Because of the complex changes in the environment, it is highly intractable to manually collect completely calibrated image pairs with and without raindrops. Unlike the uncontrollable asynchronous shooting method in [21], we elaborately synthesize raindrop images from their raindrop-free versions with a manipulatable rendering model that aims to simulate the following three scenarios: (1) the image patches seen through raindrops can be inverted and blurred to some extent; (2) a new group of raindrops will randomly stack onto the original raindrops and produce collisions and merging with other raindrops on a glass window, windscreen, or lens [13]; and (3) different raindrops on the degraded images will show various shapes, scales, distribution densities, and transparency levels, especially in bad weather. Thus, similar to [12], we first select 385 training images and 44 testing images from the training and validation sets of the Cityscapes dataset [33] as the raindrop-free images. Then, we adopt and extend the image-based raindrop generation algorithm ROLE (https://github.com/ricky40403/ROLE) to render raindrops on the 429 selected images.

**Figure 1:**
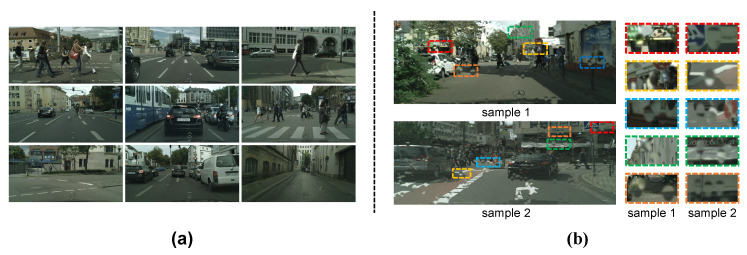
Some examples in the proposed RaindropCityscapes dataset: (**a**) examples of raindrop-free background scenes; (**b**) two raindrop image samples with diverse raindrop characteristics. Please zoom into these image samples for more details.

**Figure 2:**
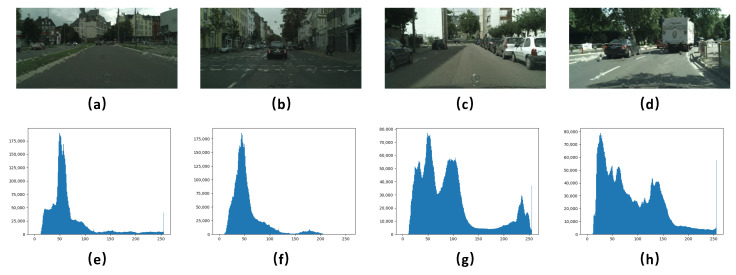
Some examples in the proposed RaindropCityscapes dataset: (**a**,**b**): two raindrop image examples in low illumination; (**c**,**d**): two raindrop image examples in high illumination; and (**e**–**h**): the histograms that reveal the intensity distribution of the raindrop images.

Specifically, given a raindrop-free image 𝒳 as the input, we first generate *n* random raindrop positions $P=\{\left(x,y\right)|\left(x_{1},y_{1}\right),\cdots ,\left(x_{n},y_{n}\right)\}$, within the image X. Then, we exploit the raindrop generation function $F_{G}$ to create *n* various raindrops centered (x,y)∈P. $F_{G}$ uses the Gaussian Blur with coefficients *b* and *m* to control the blurry ranges of raindrops and the corresponding maps, respectively, where a larger *b* or *m* means that the image patches seen through the raindrops become more blurred. To model the raindrops more realistically, the $F_{G}$ also adds a fish-eye effect to simulate and distort the occluded background. Meanwhile, most backgrounds in the raindrop can be flipped, and the sizes of all generated raindrops are constrained by the radius coefficient *r*. Following the generation stage, we apply the check function $F_{C}$ consisting of handling collision and bounding adjustments to improve the generated raindrops on the glass surfaces. The details of $F_{G}$ and $F_{C}$ are based on the algorithm ROLE. Therefore, the pipeline of raindrop rendering can be formulated as:

(1) $$F_{\left(r,n,b,m\right)}\left(X\right)=F_{C}\left(F_{G}^{\left(r,n,b,m\right)}\left(X\right)\right),$$

(2) $$I^{N+1}=F_{\left(r,n,b,m\right)}\left(I^{N}\right),N=0,1,\ldots$$

where $F_{\left(r,n,b,m\right)}\left(X\right)$ indicates the whole raindrop rendering function for a single image and *N* means the repeated rendering times for the input image *I*, which simulates a new group of raindrops. Note that $I^{0}=I$ when N=0. Here, we empirically set the max *N* to 2 and use a set of parameters {(10,14,16),(180,240,300),(0.9,1.35,1.8),(4,6,8)} for raindrop radius *r*, amounts *n*, fuzzy coefficient *b* and *m*, respectively. Moreover, to make the raindrops harmonious with the background, we combine the fuzzy coefficient *b* and *m* as joint settings. In this way, each input image will produce 27 raindrop image variants with different parameter settings. Altogether, our RaindropCityscapes dataset contains 10,395 training images and 1188 testing images. Figure 1 shows some examples of background scenes and raindrops in the RaindropCityscapes dataset. Moreover, to alleviate the problem that existing rendering models are difficult to simulate outdoor illumination accurately [18], the collected raindrop-free images are in different illumination conditions from the Cityscapes. Figure 2 shows some raindrop examples under low and high illumination conditions.

## 4. Proposed Method

Following the observations that raindrops are transparent, and their locations are randomly distributed on a single image, Ref. [21] first analyzes the raindrop image formation, which regards the raindrop degraded image I as a combination of a clean background image B and the blurry effect of the raindrops R, as follows:

(3) I=(1−M)⊙B+R,

where M indicates the binary mask. In the mask, the pixel x is part of the raindrop region if M(x)=1; otherwise, it belongs to the background region. The operator ⊙ means element-wise multiplication. Then, based on the model in Equation (3), Ref. [21] combines the raindrop image with the final estimated attention map $M_{\mathrm{att}}$ for deraining as follows:

(4) $$D=G(I,M_{\mathrm{att}}),$$

where D is the predicted derained result and G represents the contextual autoencoder network.

Instead of exploiting the generative adversarial network under the guidance of attention map to remove raindrops implicitly, we further utilize the estimated raindrop location map to explicitly separate the raindrop region from the background region and obtain the final derained result by

(5) $$D=I⊙\left(1-M_{k}\right)+T⊙M_{k},$$

where $M_{k}$ indicates the estimated raindrop location map in [0, 1] and T means the coarse derained result for I. More specifically, the pixel x is more likely to be in a raindrop region when $M_{k}\left(x\right)$ is higher i.e., $M_{k}\left(x\right)\to 1$, and vice versa. Thus, different from the image-level density label [7] without location information of rain, it can be seen that the pixel-level map operation on the raindrop image, i.e., $I⊙(1-M_{k})$, tends to preserve the original image details, especially for image regions free from the occlusion of raindrops. This can significantly reduce the risks of over-deraining [7], led by the directly residual subtraction. Meanwhile, the map operation on the coarse result, i.e., $T⊙M_{k}$, explicitly extracts the derained imagery on the occluded regions, which promotes the convolution filters to focus more on removing raindrops with diverse shapes, scales, and transparency levels.

**Figure 3:**
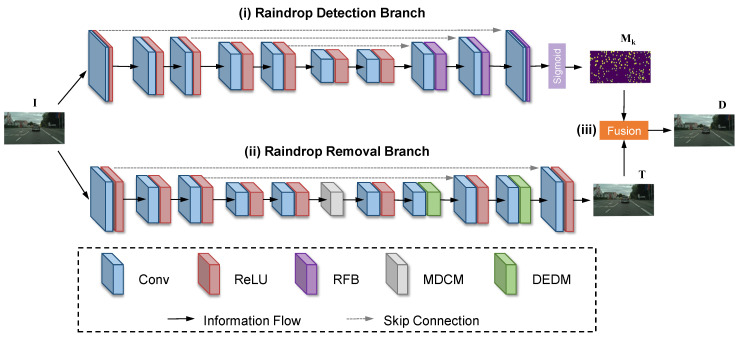
The overall architecture of the proposed MSANet: (**i**) the raindrop detection branch accurately estimates raindrop location information with a multi-path receptive field block (RFB); (**ii**) the raindrop removal branch eliminates raindrops that vary in shape, scale, and transparency with the multi-scale dilated convolution module (MDCM) and multi-scale densely enhanced deformable module (DEDM); and (**iii**) the final fusion between the coarse derained result and raindrop-free background using the raindrop location information for better deraining.

Therefore, our goal is first to estimate a raindrop location map $M_{k}$ while predicting the coarse derained result T from the given raindrop image I. By combining I and T with the guidance of $M_{k}$ as illustrated in Equation (5), we can obtain the final derained result D. In this way, we propose a novel end-to-end network for raindrop removal called the multi-scale shape adaptive network (MSANet) which employs raindrop detection and removal branch to generate $M_{k}$ and T, respectively. For clarity, the architecture of the proposed MSANet is shown in Figure 3.

### 4.1. Raindrop Detection Branch

Since the image regions occluded by raindrops are randomly distributed, it is intractable to remove raindrops while keeping the image details of the raindrop-free regions well-preserved. This problem tends to become worse for raindrops with various shapes, scales, and transparency levels, and when adjacent raindrops merge. To resolve this problem, we adopt a similar autoencoder network-based detection branch to produce the raindrop map $M_{k}$ and determine the locations of the raindrops. Furthermore, to handle raindrops with different granularity levels, we introduce the receptive field block (RFB) [34] to strengthen the discriminability of the deep raindrop features learned from the upsampling stage in Figure 3, which can effectively avoid error detection.

**Figure 4:**
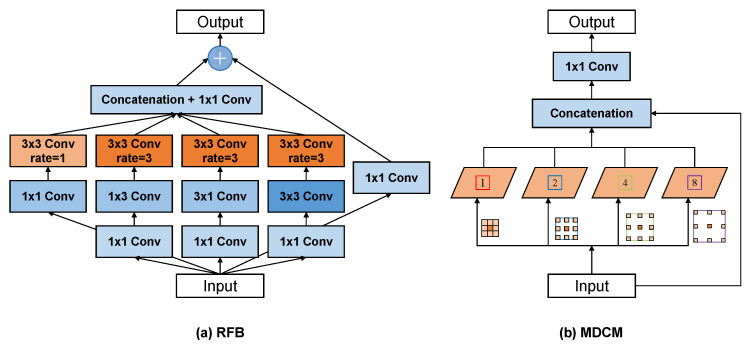
The schematic illustration of (**a**) the receptive field block (RFB) and (**b**) the multi-scale dilated convolution module (MDCM).

Specifically, the RFB contains multi-path forward convolution layers with different kernels and dilated convolution layers, as shown in Figure 4a. For the input feature maps $f_{I}\in R^{H\times W\times C}$ from the previous layer, several 1 × 1 conv-layers are first employed to decrease the number of channels. Second, instead of integrating cascaded convolution layers that use large kernel sizes (e.g., 3 × 3 and 5 × 5) as in [35], the RFB uses a combination of small irregular kernels (e.g., 1 × 3 and 3 × 1) to extract the detailed features, such as the edge information between raindrops. Meanwhile, replacing the 5 × 5 conv-layer with two stacked 3 × 3 conv-layers in some paths can reduce the number of parameters and deepen nonlinear layers in our network. Then, at the end of each path, dilated convolution is exploited to enlarge the receptive field and capture more texture information in a larger area. Lastly, the feature maps in each path are integrated together via the concatenation operation, and the output feature maps $f_{O}\in R^{H\times W\times C}$ can be obtained by additional 1 × 1 conv-layers.

### 4.2. Raindrop Removal Branch

To remove diverse raindrops with complex shapes and scale changes while restoring and preserving image details, we design a raindrop removal branch consisting of a multi-scale dilated convolution module (MDCM) and a multi-scale densely enhanced deformable module (DEDM).

#### 4.2.1. Multi-Scale Dilated Convolution Module

Influenced by the different shapes and refractive indexes, the image content seen through raindrops depends on the raindrop occluded background and the whole environment [21]. The raindrops with high transparency refractive indexes tend to produce remarkably different semantics from the occluded background, which have serious impacts on raindrop removal and are neglected in [7,10]. To address this problem, we introduce a multi-scale dilated convolution module (MDCM) in the middle junction of the encoder and decoder, as shown in Figure 3.

Because of the consecutive downsamplings in the encoder, the image features lose too much detailed texture and become coarse in raindrop boundaries. However, consistent semantics and background outlines can be found in these features and can be helpful for the restoration of image content. To fully capture the image semantics of raindrops, we utilize dilated convolution with multiple dilations to enlarge the receptive field of the raindrop removal branch. Specifically, we design different levels of dilations (e.g., 1, 2, 4, 8) as shown in Figure 4b. The feature maps from different dilated convolutions are merged together with the input feature maps, followed by a 1 × 1 convolution layer.

#### 4.2.2. Multi-Scale Densely Enhanced Deformable Module

As a standard convolution in CNN, the regular sampling location grid ℛ with a convolution kernel of 3 × 3 is defined as R={(−1,−1),(−1,0),⋯,(0,1),(1,1)}. Then, for each location $p_{0}$ on the input feature map f, the output feature map $y(p_{0})$ can be formulated as:

(6) $$y\left(p_{0}\right)=\sum_{p_{k}\in R}w\left(p_{k}\right)\cdot f\left(p_{0}+p_{k}\right),$$

where $p_{k}$ enumerates all locations in R and $w(p_{k})$ weights the sampled values at the *k*-th grid location.

**Figure 5:**
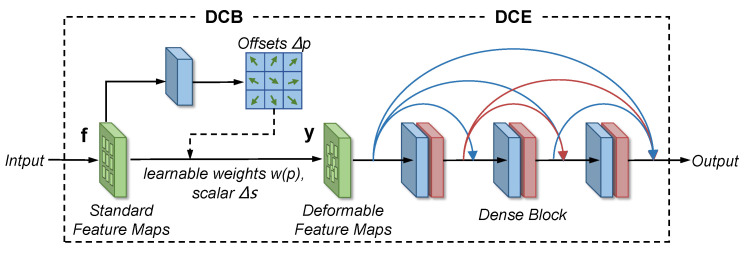
The architecture of the proposed densely enhanced deformable module (DEDM).

However, conventional sampling methods in existing deraining methods [7,18,21] are inherently limited in geometric transformation modeling [27], especially for raindrops. Different from standard sampling with fixed $p_{k}$ in normal convolutions, adaptive learnable offsets and modulation scalars are introduced to enable free form deformation of the sampling grid R. In this paper, we adopt the more deformable convolution block (DCB) in [36]. Thus, based on the standard convolution in Equation (6), the feature map $y(p_{0})$ output by the modulated deformable convolution can be expressed as follows:

(7) $$y\left(p_{0}\right)=\sum_{k\in R}w\left(p_{k}\right)\cdot f\left(p_{0}+p_{k}+\Delta p_{k}\right)\cdot \Delta s_{k},$$

where $\Delta p_{k}$ and $\Delta s_{k}$ denote the learnable offset and the modulation scalar at the *k*-th location in R, respectively. The modulation scalar $\Delta s_{k}$ lies in the range of [0, 1], while $\Delta p_{k}$ is a real number without a constrained range. Both $\Delta p_{k}$ and $\Delta s_{k}$ are predicted from the input feature map f via an additional convolution layer, as illustrated in Figure 5.

Next, the adaptive deformable features y are fed into a densely connected enhancement (DCE), which consists of several convolution blocks with dense connections [37]. Specifically, we employ a combination of a standard convolution layer with the kernel size of 3 × 3 and a ReLU layer as a basic dense block as shown in Figure 5. Moreover, to avoid the features or gradients from vanishing during backpropagation, we apply residual learning [38] for deformable features via a skip connection.

For simplicity, the combination of DCB and DCE is called the densely enhanced deformable module (DEDM). The DEDM can model single raindrops in most scales, shapes, and transparency levels. However, when collisions and mergers occur between various raindrops, the raindrop removal may tend to be worse for larger raindrops with low transparency. To address this problem, we extend the grid R to multi-scale kernel sizes (e.g., 3 × 3 and 5 × 5) of DEDM in the two upsampling stages, to adaptively represent diverse raindrops.

### 4.3. Loss Function

In order to make the final derained result more similar to the raindrop-free ground truth, we first adopt the standard $L_{1}$ loss to measure the pixel-wise reconstruction quality:

(8) $$L_{1}=\frac{1}{HWC}\sum_{h=1}^{H}\sum_{w=1}^{W}\sum_{c=1}^{C}\left|\right|D^{h,w,c}-{D_{\mathrm{gt}}}^{h,w,c}\left|\right|,$$

where D indicates the predicted derained image and $D_{\mathrm{gt}}$ is the raindrop-free ground truth. *C*, *W*, and *H* describe the number of channels, the widths, and the heights of the images, respectively. However, a model trained with only $L_{1}$ loss tends to blur the structural details in local regions. Thus, the $L_{SSIM}$ loss [39] is used to maximize the structural similarities between D and $D_{\mathrm{gt}}$ as follows:

(9) $$L_{SSIM}=1-SSIM\left(D,D_{\mathrm{gt}}\right).$$

Moreover, we exploit $L_{M}$ loss to help the model precisely identify and locate diverse raindrops:

(10) $$L_{M}=\frac{1}{HWC}\sum_{h=1}^{H}\sum_{w=1}^{W}\sum_{c=1}^{C}\left|\right|{M_{k}}^{h,w,c}-{M_{\mathrm{gt}}}^{h,w,c}{\left|\right|}^{2},$$

where $M_{k}$ is the predicted raindrop map and $M_{\mathrm{gt}}$ is the ground-truth map, which is obtained by setting the threshold for the difference between D and $D_{\mathrm{gt}}$ similar to [21]. Therefore, the total loss function is defined as follows:

(11) $$L_{total}=L_{1}+\lambda_{s}L_{SSIM}+\lambda_{m}L_{M},$$

where $\lambda_{s}$ and $\lambda_{m}$ are the weights of $L_{SSIM}$ and $L_{M}$ respectively, which are set to 0.5 and 0.1.

## 5. Experiments

### 5.1. Implementation Details

Our proposed MSANet is implemented using the framework of PyTorch [40]. During the training stage, we randomly crop the raindrop/raindrop-free image patch to a size of 256 × 512 from input image pairs of 1024 × 2048 to reduce the computational costs, which is also applied by other deraining methods in the study for a fair comparison. Furthermore, we adopt Adam [41] to optimize the network with the momentum values $\beta_{1}=0.9$ and $\beta_{2}=0.999$. The learning rate is initialized at 2 ×$10^{-4}$ and then decreased to 1 ×$10^{-5}$ after 20,000 iterations; lastly, we stop the learning after 40,000 iterations. We train the MSANet on a single NVIDIA Titan Xp GPU with a mini-batch size of 8. During the testing stage, different from [12], we directly process the rainy images at a size of 1024 × 2048 without random scaling or cropping.

### 5.2. Results and Comparisons

We conduct experiments to compare our proposed method against the state-of-the-art raindrop removal methods including Eigen [18], Pix2Pix [42], SelectGAN [43], and AGAN [21]. The deraining performance on synthetic and real-world datasets is evaluated using two metrics, the Peak Signal-to-Noise Ratio (PSNR) [44] and the Structural Similarity (SSIM) [39]. To make a fair comparison, we obtain the derained results of the compared methods by adopting either the derained outputs provided by the authors or their released models fine-tuned on the raindrop datasets.

#### 5.2.1. Comparison Results on the Synthetic Dataset

Table 1 summarizes the comparison results in terms of the PSNR and SSIM metrics. As can be observed, Ref. [18,43] have little effect on removing raindrops and even damage the texture details of images. Our proposed method considerably outperforms state-of-the-art single image deraining methods. Specifically, compared to the second best method [21], our MSANet improves the PSNR and SSIM values by an average of 2.13 db and 4.9% on the RaindropCityscapes dataset. Notably, SelectGAN [43] utilizes semantic map guidance as additional supervision data for raindrop-free image generation.

**Figure 6:**
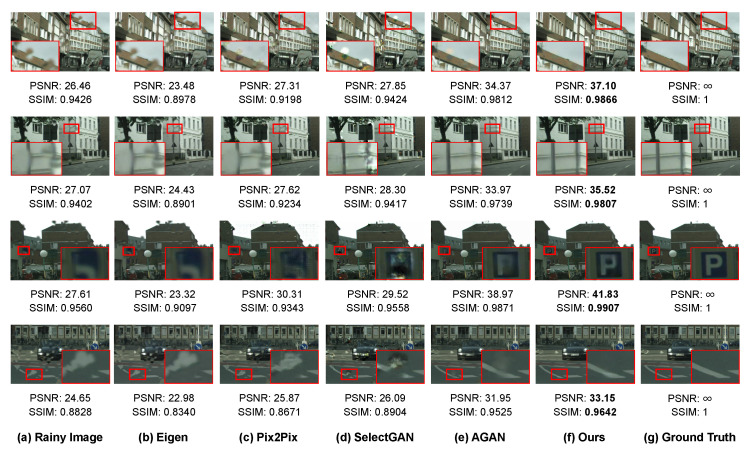
Derained results of Eigen [18], Pix2Pix [42], SelectGAN [43], AGAN [21], and our proposed MSANet on the RaindropCityscapes dataset. Please zoom into these image samples for more details.

To visually demonstrate the improvements obtained by the proposed method on the synthetic dataset, in Figure 6, we present several derained results from all the aforementioned methods. As can be seen from the eaves of the building in the 1st example result, the wall surface on the white building in the 2nd result, the letter ’P’ on the parking sign in the 3rd result, and the curved lane line on the road in the 4th result, clear differences in the effectiveness of removing raindrops with diverse characteristics and the quality of the derained images can be observed by the comparison between our proposed MSANet and the state-of-the-art methods. More specifically, for the small raindrops in the first two derained results in Figure 6, Eigen [18] hardly removes the raindrops and even blurs the reconstructed areas. Though Pix2Pix [42] and SelectGAN [43] remove more raindrops, both of them tend to leave spot artifacts and fail to recover the texture details of the background occluded by raindrops. When extended to the large raindrops in the last two results, the original image scenarios are seriously changed and distorted due to the raindrop refraction. Eigen [18], Pix2Pix [42], and SelectGAN [43] produce little effect on removing raindrops, and, compared to AGAN [21], the proposed MSANet not only removes diverse raindrops thoroughly without leaving artifacts, but also recovers and preserves more image details.

#### 5.2.2. Comparison Results on a Real-World Dataset

**Figure 7:**
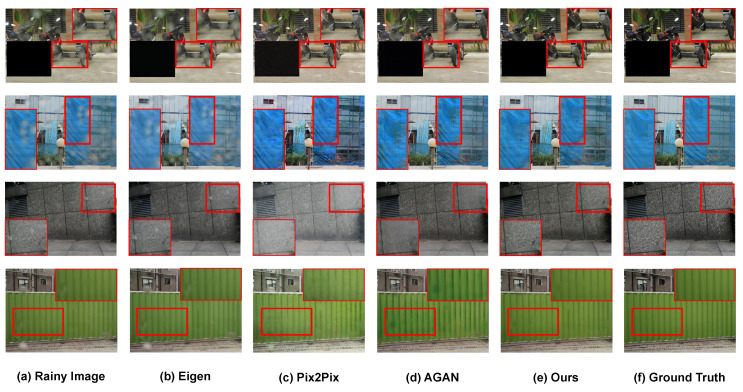
Derained results of Eigen [18], Pix2Pix [42], AGAN [21], and the proposed MSANet on the real-world raindrop dataset. Please zoom into these image samples for more details.

Similarly, to further investigate the robustness and generalization ability of the proposed method for real-world raindrop images, we compare the deraining performance of the state-of-the-art methods with our proposed MSANet on the real-world dataset collected in [21]. As depicted in Table 2, our proposed method performs better than [21] in terms of the PSNR and SSIM with improvements of 0.89 and 3.7%, respectively. We also provide some derained samples in Figure 7. As can be observed, for the dense raindrops in the first two sample images, our proposed model offers the best visual performance for raindrop removal, which is particularly useful in perfectly removing raindrops while effectively preserving image details. Meanwhile, for the few raindrops in the last two samples, our proposed method behaves well in preserving the image textures in both the raindrop and raindrop-free image regions. These results demonstrate the high generalization ability of our method for raindrop removal from a single image.

### 5.3. Ablation Study

To investigate the effectiveness of each component in our method, we perform several experiments on the synthetic raindrop dataset to compare the performance of different modules and branches.

#### 5.3.1. Effectiveness of Modules in the Raindrop Removal Branch

In the raindrop removal branch of the proposed MSANet, our method mainly involves two core modules for better raindrop removal, including a multi-scale dilated convolution module (MDCM) and a multi-scale densely enhanced deformable module (DEDM). To verify the effectiveness of the module design, we conduct a performance comparison between MSANet and its five different network variants, as shown in Table 3. For simplification, we adopt a basic encoder–decoder (ED) architecture regarded as the baseline $M_{a}$. To explore the effectiveness of multi-scale semantics feature extraction, we integrate MDCM into $M_{a}$, denoted as $M_{b}$. Because the multi-scale DEDM is composed of a deformable convolution block (DCB) and densely connected enhancement (DCE), as shown in Figure 5, $M_{c}$ first adds DCB with deformable kernels in a single size to $M_{a}$, and then DCB is extended to multi-scale DCB (MDCB) in different deformable kernel sizes, i.e., 3 × 3 and 5 × 5. Thus, $M_{d}$, $M_{e}$ and $M_{f}$ are three different experimental settings for multi-scale DEDM.

As can be observed in Table 3, each module of the removal branch improves the derained result to some extent. Specifically, the comparisons between $M_{a}$ and $M_{b}$, $M_{a}$ and $M_{c}$ show that MDCM and DCB are effective in extracting semantics features and adaptively removing raindrops with diverse shapes, respectively. Furthermore, the comparisons among $M_{d}$, $M_{e}$ and $M_{f}$ demonstrate the improvements of MDCB and DCE in enhancing the robustness against diverse raindrops and boosting deraining performance. Meanwhile, the experimental setting $M_{f}$ refers to the proposed MSANet.

**Figure 8:**
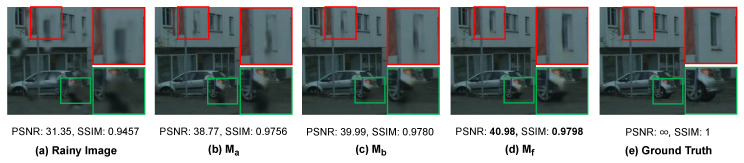
Visual quality comparison of the ablation study on modules in the raindrop removal branch. (**a**) input rainy image; (**b**–**d**): the derained results with three network settings $M_{a}$, $M_{b}$ and $M_{f}$, denoted as $M_{a}$, $M_{b}$ and $M_{f}$, respectively; and (**e**) the raindrop-free ground truth.

The raindrop removal effects of some ablation settings shown in Figure 8 are used to intuitively validate the improvements obtained by MDCM and multi-scale DEDM in $M_{f}$. In the comparison with the derained results of $M_{a}$ and $M_{b}$, it can be seen that MDCM can effectively extract texture features to remove raindrops and restore semantic information in the derained image. However, $M_{b}$ fails to process large raindrops and even produce black artifacts since the image background is seriously occluded by large raindrops. By comparing the derained results of $M_{b}$ and $M_{f}$, we can see that, after adding the multi-scale DEDM into the raindrop removal branch, more image details can be preserved clearly, and we can obtain the final derained image with improved PSNR and SSIM values.

#### 5.3.2. Effectiveness of the Raindrop Detection Branch

To delve into the improvements obtained by the raindrop detection branch, we first conduct an investigation of the proposed MSANet with and without the raindrop detection branch that does not contain the RFB module. As can be seen in Table 4, employing the raindrop detection branch improves our derained results by 0.3 db and 0.06% in terms of PSNR and SSIM, respectively, which verifies the effectiveness of our differentiated processing strategy for raindrop and raindrop-free regions. Moreover, the deraining performance of our MSANet obtains more improvements by integrating RFB into the raindrop detection branch to generate the raindrop map more accurately.

**Figure 9:**
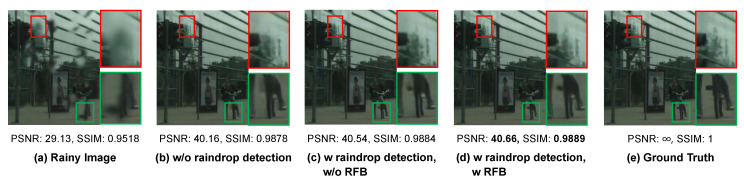
Visual quality comparison of the ablation study on branch in the proposed method: (**a**) input rainy image; (**b**–**d**) the derained results with three network settings related to the raindrop detection branch and RFB; and (**e**) the raindrop-free ground truth.

Figure 9 visualizes the derained results comparison for the ablations in the raindrop detection branch. As can be seen in Figure 9b,c, the proposed MSANet without the raindrop detection branch tends to lose some important texture details, such as treetops and bicycle pedals because it cannot precisely distinguish the raindrop regions from the raindrop-free regions. Furthermore, after Figure 9d embedding RFB into the convolution layers of Figure 9c, our MSANet effectively removes raindrops while preserving more edge details of the background, which make the derained image closer to the raindrop-free ground truth in Figure 9e.

**Figure 10:**
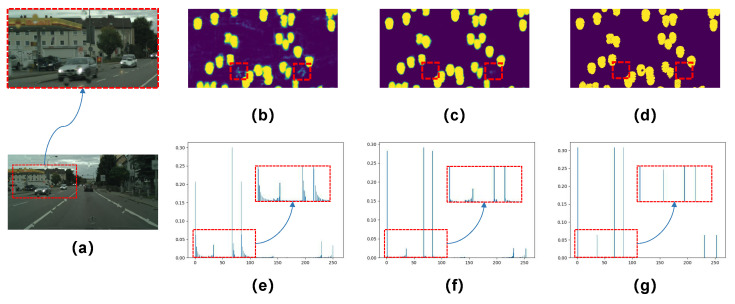
Visualization comparison of the estimated raindrop maps $M_{k}$ with and without RFB in the raindrop detection branch: (**a**) input rainy image; (**b**,**c**) the estimated raindrop maps without and with RFB, respectively; (**d**) the raindrop map ground truth; and (**e**–**g**): the normalized histograms that reveal the intensity distributions of the estimated raindrop maps, respectively.

Figure 10 further shows the estimated raindrop maps with and without RFB in the raindrop detection branch. As can be seen in Figure 10b–d, the detection branch without receptive field enhancement suffers from some uncertain or error estimations of raindrop locations. The intensity histograms of the corresponding raindrop maps also demonstrate that RFB decreases the noise interference and enhances the intensity distributions of the generated raindrop map, as shown in Figure 10e–g. Note that we apply the sigmoid activation to normalize the values of $M_{k}$ to lie in the range of [0, 1], and the raindrop map ground truth in Figure 10d is obtained by subtracting the raindrop-free ground truth from the raindrop image using a smaller threshold of zero, compared to that in [21].

### 5.4. Extension for High-Level Applications

Most high-level computer vision tasks under clear environmental scenarios have achieved great improvements for practical applications. However, the performance tends to be seriously degraded by different raindrops under complex weather conditions. This motivates us to incorporate the raindrop removal method as a form of preprocessing into high-level applications. In this paper, following [45], we introduce pre-trained models of PSPNet [46] (for semantic segmentation) and Faster R-CNN [47] (for object detection) trained on the Cityscapes dataset to perform an evaluation of segmentation and detection precision, respectively. Table 5 tabulates the accuracy of segmentation under different deraining methods on the RaindropCityscapes dataset, in terms of the mean Intersection of Union (mIoU) and mean Accuracy of each class (mAcc). Moreover, the detection precision is compared using the values of the mean Average Precision (mAP) and Average Precision at a threshold of 0.5 (AP${}_{50}$).

**Figure 11:**
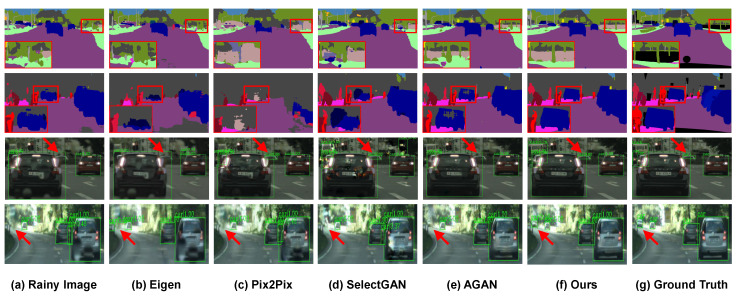
Visual comparison of semantic segmentation and object detection on the RaindropCityscapes dataset. The first two rows denote the segmentation results by PSPNet [46], and the last two rows are the detection results of Faster R-CNN [47]. More details can be observed by zooming into the figure.

It can be seen that rainy images without deraining suffer from low segmentation confidence in mIoU and mAcc since raindrops with diverse shapes, scales, and transparency levels greatly change the distribution of pixels in each class. In addition, raindrop refraction and occlusion can damage the image details and seriously reduce the detection precision. Compared to the state-of-the-art raindrop removal methods, the segmentation precision and detection accuracy of the derained results using the proposed MSANet achieves significant improvements in preserving more image details while removing diverse raindrops. We also provide more visual comparisons in Figure 11, showcasing the effectiveness of our proposed method in semantics restoration and details preservation.

### 5.5. Discussion

In order to evaluate the computational efficiency of the proposed method, we perform other recent deraining methods and our method on the same machine NVIDIA Titan Xp GPU with 12 GB memory to ensure a fair comparison. In detail, we feed 1000 testing images to the deraining network and calculate the average processing time. The list of average running time per image for different deraining methods is shown in Table 6. As can be observed, the running time of our method is competitive to AGAN [21]. Though we expend a little more running time than Pix2Pix [42] as well as SelectGAN [43], our method exceeds them with great improvements of deraining performances. Note that Eigen [18] only provides the released code on the platform of Matlab, while other methods on the Pytorch [40].

**Figure 12:**
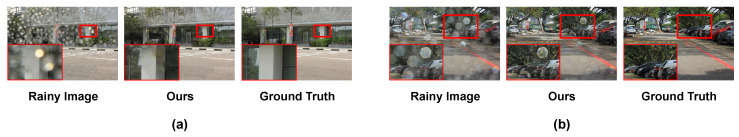
Failure cases. Our method fails to remove raindrops with some highly bright reflection artifacts in (**a**) and colorful rain-like reflection spots in (**b**).

Figure 12 shows two main types of limitation examples. The first type of limitation occurs when some highly bright reflection artifacts exists in a rainy image. For example, in Figure 12a, our method might not work well in preserving the image details, though it can remove the reflection artifacts. Another case is that our method fails to process large and colorful rain-like reflection spots due to the complexity of raindrop generation. For instance, in Figure 12b, some obvious rain-like artifacts cannot be removed effectively. One possible reason for this failure is that existing training datasets do not consider similar raindrop conditions, which contain raindrops with diverse reflection artifacts. This can be alleviated by collecting more raindrop samples in the future work.

## 6. Conclusions

In this paper, we first establish a large-scale raindrop dataset named RaindropCityscapes, consisting of 11,583 pairs of high-resolution raindrop and raindrop-free images at different scales, densities, and transparency levels. Then, we propose a two-branch Multi-scale Shape Adaptive Network (MSANet) comprised of raindrop detection and removal branches for removing raindrops from a single image. The raindrop detection branch uses the receptive field block (RFB) to strengthen raindrop feature discriminability for locating various raindrops accurately. Meanwhile the raindrop removal branch adopts the multi-scale dilated convolution module (MDCM) and multi-scale densely enhanced deformable module (DEDM) to effectively extract semantic information and adaptively remove diverse raindrops, respectively. Lastly, the final derained image is obtained by fusing the input raindrop image and the coarse derained result through the guidance of the raindrop map. Extensive experiments on both synthetic and real-world images, along with the outdoor raindrop image segmentation and detection tasks, demonstrate that our MSANet significantly outperforms recent state-of-the-art methods.

## Figures and Tables

**Table 1:** Quantitative comparison results on the synthetic RaindropCityscapes dataset.

	Rainy Image	Eigen [18]	Pix2Pix [42]	SelectGAN [43]	AGAN [21]	Ours
PSNR	30.61	25.00	31.33	30.46	38.32	**40.45**
SSIM	0.9514	0.9013	0.9302	0.9463	0.9809	**0.9857**

**Table 2:** Quantitative comparison of the results on the real-world dataset [21].

	Rainy Image	Eigen [18]	Pix2Pix [42]	AGAN [21]	Ours
PSNR	21.41	17.64	21.24	24.43	**25.32**
SSIM	0.7502	0.6128	0.6707	0.7975	**0.8270**

**Table 3:** Ablation study on different modules of the raindrop removal branch in MSANet.

Module	$M_{a}$	$M_{b}$	$M_{c}$	$M_{d}$	$M_{e}$	$M_{f}$
ED	*√*	*√*	*√*	*√*	*√*	*√*
MDCM		*√*		*√*	*√*	*√*
DCB			*√*	*√*		
MDCB					*√*	*√*
DCE						*√*
PSNR	38.45	39.41	39.63	39.87	40.12	**40.45**
SSIM	0.9814	0.9834	0.9839	0.9846	0.9850	**0.9857**

**Table 4:** Ablation study on the network branch and module of raindrop detection branch in MSANet.

Our MSANet	PSNR	SSIM
w/o raindrop detection	39.91	0.9844
w/raindrop detection, w/o RFB	40.21	0.9850
w/raindrop detection, w/RFB	**40.45**	**0.9857**

**Table 5:** Quantitative comparison results of semantic segmentation and object detection after using different raindrop removal methods on the RaindropCityscapes dataset.

Semantic Segmentation; Algorithm: PSPNet [46]						
	**Rainy Image**	**Eigen** [18]	**Pix2Pix** [42]	**SelectGAN** [43]	**AGAN** [21]	**Ours**
mIoU (%)	67.1	57.7	57.6	66.5	72.3	**73.0**
mAcc (%)	76.9	65.6	67.3	78.4	79.8	**80.6**
**Object Detection; Algorithm: Faster R-CNN** [47]						
	**Rainy Image**	**Eigen** [18]	**Pix2Pix** [42]	**SelectGAN** [43]	**AGAN** [21]	**Ours**
mAP (%)	34.9	26.5	35.1	37.5	43.4	**43.8**
AP${}_{50}$ (%)	58.1	45.9	57.3	61.4	67.2	**67.7**

**Table 6:** The average running time (seconds) per image for different deraining methods.

Image Size	Eigen [18]	Pix2Pix [42]	SelectGAN [43]	AGAN [21]	Ours
512 × 512	1.134	0.012	0.062	0.121	0.082
1024 × 1024	3.715	0.040	0.223	0.459	0.329
